# Mechanical Unloading of the Left Ventricle before Coronary Reperfusion in Preclinical Models of Myocardial Infarction without Cardiogenic Shock: A Meta-Analysis

**DOI:** 10.3390/jcm11164913

**Published:** 2022-08-21

**Authors:** Stefano Benenati, Gabriele Crimi, Andrea Macchione, Corinna Giachero, Fabio Pescetelli, Manrico Balbi, Italo Porto, Matteo Vercellino

**Affiliations:** 1 Cardiovascular Disease Chair, Department of Internal Medicine (Di.M.I.), University of Genoa, 16132 Genoa, Italy; 2 Cardiovascular Disease Unit, IRCCS Ospedale Policlinico San Martino, IRCCS Italian Cardiology Network, 16132 Genova, Italy

**Keywords:** mechanical circulatory support, myocardial infarction, left ventricular unloading, mechanical preconditioning, infarct size

## Abstract

Aim: to compare a conventional primary reperfusion strategy with a primary unloading approach before reperfusion in preclinical studies. Methods: we performed a meta-analysis of preclinical studies. The primary endpoint was infarct size (IS). Secondary endpoints were left ventricle end-diastolic pressure (LVEDP), mean arterial pressure (MAP), heart rate (HR), cardiac output (CO). We calculated mean differences (MDs) and associated 95% confidence intervals (CIs). Sensitivity and subgroup analyses on the primary and secondary endpoints, as well as a meta-regression on the primary endpoint using the year of publication as a covariate, were also conducted. Results: 11 studies (*n* = 142) were selected and entered in the meta-analysis. Primary unloading reduced IS (MD −28.82, 95% CI −35.78 to −21.86, I^2^ 96%, *p* < 0.01) and LVEDP (MD −3.88, 95% CI −5.33 to −2.44, I^2^ 56%, *p* = 0.02) and increased MAP (MD 7.26, 95% CI 1.40 to 13.12, I^2^ 43%, *p* < 0.01) and HR (MD 5.26, 95% CI 1.97 to 8.55, I^2^ 1%, *p* < 0.01), while being neutral on CO (MD −0.11, 95% CI −0.95 to 0.72, I^2^ 88%, *p* = 0.79). Sensitivity and subgroup analyses showed, overall, consistent results. The meta-regression on the primary endpoint demonstrated a significant influence of the year of publication on effect estimate. Conclusions: in animal models of myocardial infarction, a primary unloading significantly reduces IS and exerts beneficial hemodynamic effects compared to a primary reperfusion.

## 1. Introduction

In patients with ST segment elevation myocardial infarction (STEMI), prompt restoration of blood vessel patency through percutaneous coronary intervention (PCI) limits infarct size (IS), thereby reducing the long-term risk of death and heart failure [1]. Grounded on the evidence of a time-dependent benefit of reperfusion, contemporary guidelines emphasize a 90 min cut-off from STEMI diagnosis to culprit lesion crossing with coronary wire [2]. Efforts to further shorten this delay, however, have not resulted in a consistent reduction of mortality [3]. 

It is nowadays acknowledged that reperfusion itself contributes to myocardial damage by promoting thrombus embolization, reactive oxygen species spreading, and inflammation within the viable myocardium (among others, pathophysiological drivers of the so called ischemia-reperfusion injury, IRI) [4]. On the other hand, accumulating evidences from preclinical models suggest that unloading the left ventricle before restoring coronary flow—even when reperfusion is purposely delayed—promotes the activation of cardioprotective pathways and limits IS [5,6].

From a pathophysiological standpoint, such a “primary unloading” approach stems from the association between IS and oxygen consumption [7]. During ischemia, indeed, the latter heightens as a result of the compensatory increase of myocardial contractility and heart rate (HR), producing a detrimental spiral which ultimately boosts the propagation of the necrosis. As selected mechanical circulatory supports (MCSs), have the capability of blunting myocardial work and oxygen consumption, they counteract this vicious circle and are thought to reduce the final IS [7]. 

Of note, despite MCSs being an established option to supplement cardiac output and unload the left ventricle in STEMI presenting with cardiogenic shock [8], their use in hemodynamically stable patients remains far from routine clinical practice. Although globally encouraging, the results of preclinical studies in this field have been, indeed, inconsistent, hampering a wider application of such an approach. Furthermore, whilst previous meta-analyses of animal studies have already proven the benefit of MCSs on IS [9], none has specifically addressed the effect of a primary unloading approach with or without delayed reperfusion.

The present work aims to fill this gap by comparing the primary unloading approach to the current gold standard of primary reperfusion in preclinical models of myocardial infarction without cardiogenic shock.

## 2. Methods

### 2.1. Study Selection, Eligibility Criteria and Risk of Bias 

The work has been conducted according to the Cochrane Collaboration and Preferred Reporting Items for Systematic Reviews and Meta-Analysis (PRISMA) guidelines [10] and is registered on PROSPERO (CRD42022271799, Table S1). 

We included studies conducted on preclinical (animal) models of myocardial infarction in which (1) a primary unloading strategy, defined as LV unloading before the onset or reperfusion, was compared with a primary reperfusion one, intended as a reperfusion strategy without LV unloading; and (2) LV unloading was obtained by means of a MCS. No design restriction was applied. We excluded studies reporting no sufficient data for outcome analysis or in which outcomes definition was not compliant with that chosen for the primary analysis (see below), with no retrievable full text and written in languages other than English. Likewise, duplicates were not included. In the case of multi-arm studies, all arms adopting a primary unloading strategy were assessed for eligibility and, in case, compared with the same control group. When the study protocol foresaw the use of multiple MCS, we elected to include the data of the arms treated with the MCS primarily intended as an unloading device (e.g., data from the group treated with the Impella pump were chosen instead of those of the extra-corporeal membrane oxygenation—ECMO—group). 

Between March and July 2021, a systematic digital search was performed in MEDLINE/PubMed, Cochrane and Embase. The following search terms were applied and combined according to the strategy reported in the Table S2: “left ventricular unloading”, “mechanical circulatory support”, “myocardial infarction”, “mechanical preconditioning”, and “primary unloading”. Reference lists of included articles were screened using a snowball approach. Two independent investigators (SB and MV) screened titles and abstracts to assess the eligibility. Full-texts, Supplementary Materials, online appendices, and reference lists of the eligible articles were thus examined to evaluate whether they met the prespecified inclusion/exclusion criteria. Consensus was reached in case of disagreement. The quality of the studies and risk of bias were assessed using the SYstematic Review Centre for Laboratory animal Experimentation (SYRCLE) tool [11]. For each study, we collected data regarding the enrolled animals, the protocol of ischemia/reperfusion, the type of MCS, the methodology to assess infarct size and the measurements collected during the experiment.

### 2.2. Endpoint Definitions

The primary endpoint was infarct size measured as the percentage of the area at risk. Secondary endpoints were LV end-diastolic pressure (LVEDP), mean arterial pressure (MAP), heart rate (HR), cardiac output (CO). Endpoints definitions were borrowed from each study protocol.

### 2.3. Statistical Analysis

Mean differences (MDs) between the experimental and the control arm and associated 95% confidence intervals (CIs) were calculated for each endpoint. The Higgins I^2^ statistics was used to estimate the between-studies heterogeneity, with I^2^ < 25%, 25–50%, or >50% respectively indicating low, moderate or high heterogeneity [12]. Results of the random-effect model with inverse variance weighting were considered in case of moderate-to-high heterogeneity, otherwise results of the Mantel-Haenszel fixed-effect model were reported. 

Sensitivity analyses were carried out excluding one study at time (leave-one-out approach) and including only studies in which the Impella pump was used. Furthermore, to expand the generalizability of our result, a sensitivity analysis on the primary endpoint was also ran including studies in which IS definition did not meet the criteria for inclusion (i.e., was assessed as the percentage of infarcted myocardium relative to the whole muscular mass or was just unclear) [6,13,14,15,16]. To evaluate the impact of delayed reperfusion on the primary outcome, we also performed a post-hoc subgroup analysis dichotomizing the studies according to whether such a strategy had been followed or not.

Finally, a meta-regression for the primary endpoint was also conducted to explore the impact of the year of publication on effect size. Analyses were performed pooling study-level data by means of the R software (R Foundation for Statistical Computing, version 3.6.1, Vienna, Austria).

## 3. Results

Among 2048 articles initially screened, 11 studies were selected and pooled in the meta-analysis (Figure S1) [5,17,18,19,20,21,22,23,24,25,26]. Overall, 142 animals were enrolled, 79 (56%) being adult swine. As for MCSs types, 7/11 (63%) studies adopted the Impella pump (Impella, Abiomed, Danvers, MA, USA), whereas intra-aortic balloon pump (IABP), left atrial to femoral artery (LA-FA) bypass, A-Med left ventricular assist device (LVAD) (A-Med Systems, West Sacramento, Calif) and TV LVAD (Medtronic, Minneapolis, MN, USA) were used in one case each. Of note, reperfusion was purposely delayed in the experimental arm in three protocols. The study protocols and the methods to achieve coronary ischemia are described in Table 1. The quality of the studies was overall moderate, primarily due to the lack of randomization processes (Figure S3).

**Figure 1:**
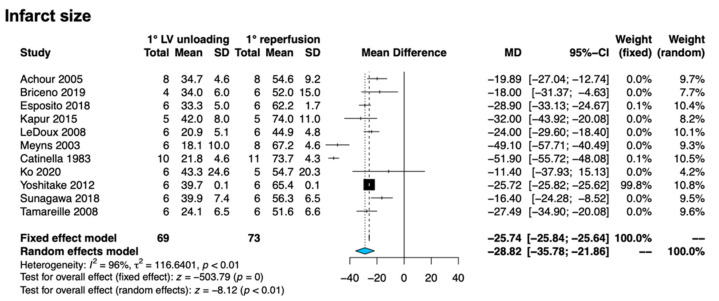
Forest plot for infarct size. Abbreviations: CI: confidence interval; LV: left ventricle; MD: mean difference; SD: standard deviation. References: Achour et al. [17], Briceno et al. [18], Esposito et al. [5], Kapur et al. [20], LeDoux et al. [22], Meyns et al. [23], Catinella et al. [19], Ko et al. [21], Yoshitake et al. [26], Sunagawa et al. [24], Tamareille et al. [25].

IS measured as a percentage of the area at risk (IS/AAR) was quantified in all studies, and consistently assessed through histopathology (using triphenyltetrazolium chloride—TTC—staining) in the acute phase. Compared to the primary reperfusion strategy, the primary unloading one resulted in a significant reduction of IS (MD −28.82, 95% CI −35.78 to −21.86, I^2^ 96%, *p* < 0.01), as shown in Figure 1. This finding was substantially unaffected by individual study removal (Figure S2) and was also preserved after restricting the analysis to studies adopting the Impella pump only (Figure S3). The explorative subanalysis including studies that did not comply with the original inclusion criteria (i.e., in which IS was defined as a percentage of the total myocardium or de definition was unclear) yielded results comparable with the main analysis, notwithstanding a significant quantitative difference among subgroups (other definition of IS: MD −12.73, 95% CI −20.70 to −4.77, I^2^ 90%, *p* < 0.01, *p* for subgroup difference < 0.01 Figure S4). Importantly, the subanalysis exploring the impact of delayed reperfusion revealed no significant differences among subgroups (Figure S5).

The meta-regression according to the year of publication showed larger effect sizes in older studies (intercept = −1747.61, beta coefficient = 0.86, *p* < 0.01, Figure S6). The funnel plot showed hints of publication bias in favor of the unloading strategy (Figure S7).

**Figure 2:**
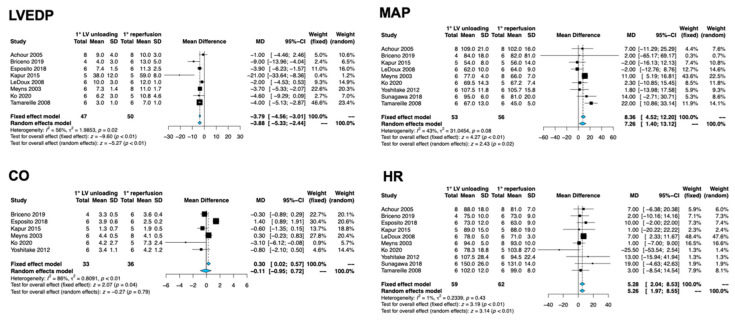
Forest plots for the secondary endpoints; Abbreviations: CI: confidence interval; CO: cardiac output; HR: heart rate; LV: left ventricle; LVEDP: LV end-diastolic pressure; MAP: mean arterial pressure; MD: mean difference; SD: standard deviation. References: Achour et al [17], Briceno et al. [18], Esposito et al. [5], Kapur et al. [20], LeDoux et al. [22], Meyns et al. [23], Ko et al. [21], Yoshitake et al. [26], Sunagawa et al. [24], Tamareille et al. [25].

Results for the secondary endpoints are reported in Figure 2. The primary unloading strategy significantly blunted LVEDP (MD −3.88, 95% CI −5.33 to −2.44, I^2^ 56%, *p* = 0.02) and increased MAP (MD 7.26, 95% CI 1.40 to 13.12, I^2^ 43%, *p* < 0.01) and HR (MD 5.26, 95% CI 1.97 to 8.55, I^2^ 1%, *p* < 0.01), while being neutral on CO (MD −0.11, 95% CI −0.95 to 0.72, I^2^ 88%, *p* = 0.79). After removal of individual studies, the results were substantially unmodified across all endpoints, with the exception of MAP and HR (Figure S2). In both the latter cases, the effect extent and direction were nearly preserved after all individual exclusions, although statistical significance was lost after removing the study from Meyns et al. [23] for MAP and LeDoux et al. [22] for HR. Restricting the analyses to studies adopting the Impella pump, only seven studies were finally included. Contrarily to the main analysis, LVEDP and HR resulted unaffected by primary unloading (Figure S3). Funnel plots are reported in the supplements, with the main concern of publication bias observable for MAP and an acceptable symmetry for other endpoints (Figure S7).

## 4. Discussion

We quantitatively summarized the evidence of 11 studies of primary LV unloading versus primary reperfusion in preclinical models. We found that, compared to a primary reperfusion strategy, primary unloading reduces IS (even when reperfusion is intentionally delayed) and LVEDP and increases MAP (and, only marginally, HR). No significant influence of LV unloading was instead observed on CO, which remained comparable in both arms. We also noted a significant influence of the year of publication on the extent of IS/AAR reduction, with a larger effect size in older studies.

MCSs are nowadays part of the routine practice in case of cardiogenic shock [27] and high-risk PCI [28], settings in which they ensure systemic and coronary perfusion by supporting/replacing, to a various extent, the physiological systolic output. In addition, a number of contemporary MCSs are able to relieve LV pressures and volumes reducing myocardial work and, thereby, oxygen consumption [29,30]. 

Indeed, myocardial IS decreases when MCSs are implanted in animals with myocardial infarction [9]. Specifically, LeDoux et al. first showed that LV unloading with IABP was associated with significant IS reduction only when the support was activated before reperfusion, and not thereafter [22], paving the way for subsequent studies focused on such “mechanical preconditioning”. The counterpulsation to reduce infarct size pre-PCI acute myocardial infarction (CRISP AMI) was the first randomized controlled trial (RCT) to attempt a clinical translation of mechanical conditioning with IABP in patients with STEMI not in cardiogenic shock, in which the device was inserted before reperfusion and maintained active for 12 h. Although no benefit on IS was noted, the trial was inherently limited by the modest hemodynamic effect of IABP as well as the noticeable time-interval from symptoms onset to the first device therapy (>3 h in each arm) [31].

In 2013, Kapur et al. showed that an unloading period of 30 min before reperfusion could increase the activation of the reperfusion injury salvage kinase (RISK) pathway, which is known to promote cardio-protection and limit apoptosis, ultimately reducing the extension of the necrosis [6]. Moreover, in a multi-arm study on adult swine undertaken by Esposito and colleagues, activating the Impella pump 30 min before reperfusion yielded the largest IS reduction, even when compared to an activation within 15 min [5]. In both studies, the greater effect of primary unloading was therefore achieved when reperfusion was significantly postponed with respect to MCS activation [5,6]. In addition, both protocols made use of MCSs such as the LA-FA bypass or the Impella pump, which overcome some limitations of IABP providing a more powerful hemodynamic support. Taken together, these findings supported the protective effect of an appropriately lasting and powerful mechanical unloading, and provided clues that delaying reperfusion may not be detrimental, but rather beneficial, as unloading meanwhile promotes antiapoptotic signals and protects mitochondrial integrity, counteracting IRI. 

Several therapies have been proposed as adjunct strategies in patients with STEMI. Examples include remote ischemic conditioning, adenosine infusion, therapeutic hypothermia, pressure-controlled intermittent coronary sinus occlusion and administration of supersaturated oxygen [32]. Efficacy of the aforementioned methods remains matter of debate and clinical translation has been poor so far, mirroring the paucity of available evidences. Importantly, as opposite to pharmacological and physical approaches of preconditioning, the one based on LV unloading through MCSs ensures systemic hemodynamics support while waiting for reperfusion, stabilizing the patient and allowing to gain precious time to properly “prepare” the heart to reperfusion itself. 

Published in 2019, the Door-To-Unload in STEMI (DTU-STEMI) pilot trial randomized 50 humans with anterior STEMI to LV unloading with Impella CP + reperfusion delayed by 30 min versus a timely primary PCI. This study confirmed that primary unloading is feasible and—as long as temporal cut-off to be effective are respected—does not curtail the benefit of timely reperfusion [33], grounding the basis for the currently ongoing STEMI Door-to-Unload (STEMI-DTU) RCT (NCT03947619), which is finally testing this strategy in a large human population of selected anterior STEMI. 

Our results expand on this evidence, confirming the beneficial effect of mechanical preconditioning on IS and providing further insights into the hemodynamic effect of MCS in preclinical models of myocardial infarction. In particular, our subanalysis exploring IS differences according to the time of reperfusion showed no significant variations of the effect among subgroups, reinforcing the concept that delaying reperfusion is not detrimental when mechanical support has been properly started. This finding is key, as the main barrier toward the clinical translation of the primary unloading approach remains a safety one: the fear of wasting precious time for reperfusion. The latter concept is indeed mirrored by current guidelines, which strongly emphasize the concept of fast referral to PCI-capable centers and rapid reperfusion whenever possible [2]. Therefore, temporary interruption of the revascularization strategy in favor of a primary unloading approach would definitively require a significant paradigm shift. On the other hand, in line with preliminary findings obtained on human subjects [33], our results support the potential advantages of this new approach.

We also found a significant influence of the year of publication on effect estimates of IS. We cannot provide univocal interpretations for this observation. However, a potential explanation resides in the earlier starting of LV unloading in older studies [e.g., in the studies by Meyns et al. [23] LA-FA bypass was activated 15 min after left anterior descending artery ligation, therefore unloading was longer compared to other protocols].

## 5. Limitations

We acknowledge several limitations of our study. Firstly, the present was a study-level meta-analysis, therefore accounting for baseline differences among the enrolled animals was substantially impossible. Our population encompasses different species and study protocols, which might have influenced the final results. In this regard, however, overall bias should be limited given that the outcomes of interest were consistently assessed with the same approaches (i.e., invasive measurements and surface ECG for CO, MAP, HR and LVEDP and histopathology for IS) across studies. However, differences in terms of unloading protocol (e.g., duration and power of support) might have significantly influenced the study results. Unfortunately, a precise characterization of the effects of LV unloading duration and power on the explored outcomes was hardly possible, given the important heterogeneity of the protocols. The studies span a wide temporal timeframe; the impact of the latter on effect estimates, however, was explored by means of a meta-regression. Although all studies reported the data for the primary outcome, there were many missing data across the remaining endpoint. Finally, many studies were of modest quality due to the lack of randomization and significant publication bias existed across different endpoints.

## 6. Conclusions

In conclusion, our meta-analysis of preclinical studies supports LV unloading before reperfusion as a promising strategy to reduce IS without any safety trade-off in case of delayed reperfusion. Future investigations are needed to further explore the clinical relevance of this approach on human subjects.

## Figures and Tables

**Table 1:** Main features of the included studies.

Study	Animals	Method of Ischemia	Protocol of Ischemia	Time of MCS Activation	MCS	Delayed Reperfusion	IS Assessment
Achour 2005 [17]	Mongrel dogs	LAD ligation	Ischemia: 120 min Reperfusion: 240 min	Last 15 min of ischemia	TV LVAD	No	Histopathology (TTC staining)
Briceno 2019 [18]	Yorkshire swine	Balloon inflation into the LAD	Ischemia: 120 min Reperfusion: 180 min	Last 30 min of ischemia	Impella CP	No	Histopathology (TTC staining)
Esposito 2018 [5]	Yorkshire swine	Stent inflation into the LAD	Ischemia: 90 min (control arm) vs. 120 min (unloading arm)Reperfusion: 120 min	Last 30 min of ischemia	Impella CP	Yes (30 min)	Histopathology (TTC staining)
Kapur 2015 [20]	Yorkshire swine	Stent inflation into the LAD	Ischemia: 90 min (control arm) vs. 150 min Reperfusion: 120 min	Last 60 min of ischemia	Impella CP	Yes (60 min)	Histopathology (TTC staining)
LeDoux 2008 [22]	Yorkshire swine	LAD ligation	Ischemia: 60 min Reperfusion: 240 min	Last 15 min of ischemia	IABP	No	Histopathology (TTC staining)
Meyns 2003 [23]	Dorset sheep	Ligation of the two major diagonal branches of the LAD	Ischemia: 60 min Reperfusion: 120 min	From the onset if ischemia	Impella LV	No	Histopathology (TTC staining)
Catinella 1983 [19]	Mongrel dogs	LAD ligation	Ischemia: 240 min Reperfusion: NA	After 15 min of ischemia	LA-FA bypass	No	Histopathology (TTC staining)
Ko 2020 [21]	Yorkshire swine	Ligation of the LAD	Ischemia: 60 min (control arm) vs. 90 min (unloading arm)Reperfusion: 120 min	Last 30 min of ischemia	Impella CP	Yes (30 min)	Histopathology (TTC staining)
Yoshitake 2012 [26]	Landrace, Large White, and Duroc (LWD) swine	Ligation of the LAD	Ischemia: 120 min Reperfusion: 240 min	Last 30 min of ischemia	Impella LD	No	Histopathology (TTC staining)
Sunagawa 2018 [24]	Mongrel dogs	Ligation of the left circumflex	Ischemia: 180 min Reperfusion: 180 min	Last 120 min of ischemia	Impella CP	No	Histopathology (TTC staining)
Tamareille 2008 [25]	Yorkshire swine	Ligation of the LAD (distal third)	Ischemia: 60 minReperfusion: 240 min	Last 15 min of ischemia	A-Med LVAD	No	Histopathology (TTC staining)

Delayed reperfusion was intended as an intentional abstention from reperfusion maneuvers once left ventricular unloading had been started, prolonging the duration of the ischemic phase. Abbreviations: IABP: intra-aortic balloon pump; LA-FA: left atrium-femoral artery; LVAD: left ventricular assist device; MCS: mechanical circulatory support; TV-LVAD: transvalvular left ventricular assist device; TTC: triphenyltetrazolium chloride.

## Data Availability

Database and codes would be transmitted upon reasonable request to the Corresponding Author.

## Supplementary Materials

The following supporting information can be downloaded at: https://www.mdpi.com/article/10.3390/jcm11164913/s1, Table S1. PRISMA checklist. Table S2. Full electronic search in MEDLINE/Pubmed database through 10 July 2020. Table S3. Bias assessment. Figure S1. PRISMA flowchart. Figure S2. Sensitivity analysis with the leave-one-out approach. Figure S3. Sensitivity analysis including only studies with the Impella pump. Figure S4. Subanalysis including studies that adopted different definitions of infarct size. Figure S5. Subanalysis according to the time of reperfusion. Figure S6. Meta-regression using the year of publication as a covariate. Figure S7. Funnel plots.

## References

[1] Stone G.W., Selker H.P., Thiele H., Patel M.R., Udelson J.E., Magnus Ohman R., Maehara A., Eitel I., Granger C.B., Jenkins P.L. (2016). Relationship between Infarct Size and Outcomes Following Primary PCI: Patient-Level Analysis from 10 Randomized Trials. J. Am. Coll. Cardiol..

[2] Ibanez B., James S., Agewall S., Antunes M.J., Bucciarelli-Ducci C., Bueno H., Caforio A.L.P., Crea F., Goudevenos J.A., Halvorsen S. (2018). 2017 ESC Guidelines for the management of acute myocardial infarction in patients presenting with ST-segment elevation: The Task Force for the management of acute myocardial infarction in patients presenting with ST-segment elevation of the European Society of Cardiology (ESC). Eur. Heart J..

[3] Menees D.S., Gurm H.S. (2014). Door-to-balloon time and mortality. N. Engl. J. Med..

[4] Inserte J., Hernando V., Garcia-Dorado D. (2012). Contribution of calpains to myocardial ischaemia/reperfusion injury. Cardiovasc. Res..

[5] Esposito M.L., Zhang Y., Qiao X., Reyelt L., Paruchuri V., Schnitzler G.R., Morine K.J., Annamalai S.K., Bogins C., Natov P.S. (2018). Left Ventricular Unloading before Reperfusion Promotes Functional Recovery after Acute Myocardial Infarction. J. Am. Coll. Cardiol..

[6] Kapur N.K., Paruchuri V., Urbano-Morales J.A., Mackey E.E., Daly G.H., Qiao X., Pandian N., Perides G., Karas R.H. (2013). Mechanically unloading the left ventricle before coronary reperfusion reduces left ventricular wall stress and myocardial infarct size. Circulation.

[7] Kapur N.K., Reyelt L., Swain L., Esposito M., Qiao X., Annamalai S., Meyns B., Smalling R. (2019). Mechanical Left Ventricular Unloading to Reduce Infarct Size during Acute Myocardial Infarction: Insight from Preclinical and Clinical Studies. J. Cardiovasc. Transl. Res..

[8] Benenati S., Vercellino M., Avenoso D., Crimi G., Macchione A., Giachero C., Balbi M., Della Bona R., Gnecco G., Baronetto A. (2021). Management of cardiogenic shock: A proposal for a shared protocol. G Ital. Cardiol..

[9] Miyashita S., Kariya T., Yamada K.P., Bikou O., Tharakan S., Kapur N.K., Ishikawa K. (2021). Left Ventricular Assist Devices for Acute Myocardial Infarct Size Reduction: Meta-analysis. J. Cardiovasc. Transl. Res..

[10] Moher D., Shamseer L., Clarke M., Ghersi D., Liberati A., Petticrew M., Shekelle P., Stewart L.A. (2015). Preferred reporting items for systematic review and meta-analysis protocols (PRISMA-P) 2015 statement. Syst. Rev..

[11] Hooijmans C.R., Rovers M.M., de Vries R.B.M., Leenaars M., Ritskes-Hoitinga M., Langendam M.W. (2014). SYRCLE’s risk of bias tool for animal studies. BMC Med. Res. Methodol..

[12] Higgins J.P., Altman D.G., Gotzsche P.C., Jüni P., Moher D., Oxman A.D., Savović J., Schulz K.F., Weeks L., Sterne J.A.C. (2011). The Cochrane Collaboration’s tool for assessing risk of bias in randomised trials. BMJ.

[13] Sun X., Li J., Zhao W., Lu S., Guo C., Lai H., Wang C. (2016). Early Assistance with Left Ventricular Assist Device Limits Left Ventricular Remodeling After Acute Myocardial Infarction in a Swine Model. Artif. Organs.

[14] Saku K., Kakino T., Arimura T., Sunagawa G., Nishikawa T., Sakamoto T., Kishi T., Tsutsui H., Sunagawa K. (2018). Left Ventricular Mechanical Unloading by Total Support of Impella in Myocardial Infarction Reduces Infarct Size, Preserves Left Ventricular Function, and Prevents Subsequent Heart Failure in Dogs. Circ. Hear. Fail..

[15] Wouters P.F., Sukehiro S., Mollhoff T., Hendrikx M., Waldenberger F.R., Wiebalck A., Flameng W. (1993). Left ventricular assistance using a catheter-mounted coaxial flow pump (Hemopump) in a canine model of regional myocardial ischaemia. Eur. Hear. J..

[16] Haston H.H., McNamara J.J. (1979). The effects of intraaortic balloon counterpulsation on myocardial infarct size. Ann. Thorac. Surg..

[17] Achour H., Boccalandro F., Felli P., Amirian J., Uthman M., Buja M., Smalling R.W. (2005). Mechanical left ventricular unloading prior to reperfusion reduces infarct size in a canine infarction model. Catheter. Cardiovasc. Interv..

[18] Briceno N., Annamalai S.K., Reyelt L., Crowley P., Qiao X., Swain L., Pedicini R., Foroutanjazi S., Jorde L., Yesodharan G. (2019). Left Ventricular Unloading Increases the Coronary Collateral Flow Index before Reperfusion and Reduces Infarct Size in a Swine Model of Acute Myocardial Infarction. J. Am. Hear. Assoc..

[19] Catinella F.P., Cunningham J.N., Glassman E., Laschinger J.C., Baumann F.G., Spencer F.C. (1983). Left atrium-to-femoral artery bypass: Effectiveness in reduction of acute experimental myocardial infarction. J. Thorac. Cardiovasc. Surg..

[20] Kapur N.K., Qiao X., Paruchuri V., Morine K.J., Syed W., Dow S., Shah N., Pandian N., Karas R.H. (2015). Mechanical Pre-Conditioning with Acute Circulatory Support before Reperfusion Limits Infarct Size in Acute Myocardial Infarction. JACC Heart Fail..

[21] Ko B., Drakos S.G., Ibrahim H., Kang T.S., Thodou A., Bonios M., Taleb I., Welt F.G. (2020). Percutaneous Mechanical Unloading Simultaneously with Reperfusion Induces Increased Myocardial Salvage in Experimental Acute Myocardial Infarction. Circ. Heart Fail..

[22] LeDoux J.F., Tamareille S., Bs P.R.F., Amirian J., Smalling R.W. (2008). Left ventricular unloading with intra-aortic counter pulsation prior to reperfusion reduces myocardial release of endothelin-1 and decreases infarction size in a porcine ischemia-reperfusion model. Catheter. Cardiovasc. Interv..

[23] Meyns B., Stolinski J., Leunens V., Verbeken E., Flameng W. (2003). Left ventricular support by catheter-mounted axial flow pump reduces infarct size. J. Am. Coll. Cardiol..

[24] Sunagawa G., Saku K., Arimura T., Nishikawa T., Mannoji H., Kamada K., Abe K., Kishi T., Tsutsui H., Sunagawa K. (2019). Mechano-chronotropic Unloading during the Acute Phase of Myocardial Infarction Markedly Reduces Infarct Size via the Suppression of Myocardial Oxygen Consumption. J. Cardiovasc. Transl. Res..

[25] Tamareille S., Achour H., Amirian J., Felli P., Bick R.J., Poindexter B., Geng Y.J., Barry W.H., Smalling R.W. (2008). Left ventricular unloading before reperfusion reduces endothelin-1 release and calcium overload in porcine myocardial infarction. J. Thorac. Cardiovasc. Surg..

[26] Yoshitake I., Hata M., Sezai A., Unosawa S., Wakui S., Kimura H., Nakata K., Hata H., Shiono M. (2012). The effect of combined treatment with Impella((R)) and landiolol in a swine model of acute myocardial infarction. J. Artif. Organs.

[27] Benenati S., Toma M., Canale C., Vergallo R., Bona R.D., Ricci D., Canepa M., Crimi G., Santini F., Ameri P. (2021). Mechanical circulatory support in patients with cardiogenic shock not secondary to cardiotomy: A network meta-analysis. Heart Fail. Rev..

[28] Burzotta F., Russo G., Ribichini F., Piccoli A., D’Amario D., Paraggio L., Previ L., Pesarini G., Porto I., Leone A.M. (2019). Long-Term Outcomes of Extent of Revascularization in Complex High Risk and Indicated Patients Undergoing Impella-Protected Percutaneous Coronary Intervention: Report from the Roma-Verona Registry. J. Interv. Cardiol..

[29] Uriel N., Sayer G., Annamalai S., Kapur N.K., Burkhoff D. (2018). Mechanical Unloading in Heart Failure. J. Am. Coll. Cardiol..

[30] Suga H. (1979). Total mechanical energy of a ventricle model and cardiac oxygen consumption. Am. J. Physiol..

[31] Patel M.R., Smalling R.W., Thiele H., Barnhart H.X., Zhou Y., Chandra P., Chew D., Cohen M., French J., Perera D. (2011). Intra-aortic balloon counterpulsation and infarct size in patients with acute anterior myocardial infarction without shock: The CRISP AMI randomized trial. JAMA.

[32] Kloner R.A., Creech J.L., Stone G.W., O’Neill W.W., Burkhoff D., Spears J.R. (2021). Update on Cardioprotective Strategies for STEMI: Focus on Supersaturated Oxygen Delivery. JACC Basic Transl. Sci..

[33] Kapur N.K., Alkhouli M., DeMartini T.J., Faraz H., George Z.H., Goodwin M.J., Hernandez-Montfort J.A., Iyer V.S., Josephy N., Kalra S. (2019). Unloading the Left Ventricle before Reperfusion in Patients with Anterior ST-Segment-Elevation Myocardial Infarction. Circulation.

