# Supplementary material for: Mechanical Unloading of the Left Ventricle before Coronary Reperfusion in Preclinical Models of Myocardial Infarction without Cardiogenic Shock: A Meta-Analysis
Source: J Clin Med. 2022 Aug 21;11(16):4913. doi: 10.3390/jcm11164913 (PMC9409839; doi:10.3390/jcm11164913)
Supplement: Supplementary file 1 [file jcm-11-04913-s001.zip › jcm-1819251-supplementary.pdf]

Mechanical Unloading of the Left Ventricle Before Coronary Reperfusion in Preclinical Models of Myocardial Infarction without Cardiogenic Shock: A Meta-Analysis

S. Benenati et al.

Supplementary Materials

Table S1. PRISMA checklist.

Table S2. Full electronic search in MEDLINE/Pubmed database through 10 July 2020.

Figure S1. PRISMA flowchart.

Table S3. Bias assessment.

Figure S2. Sensitivity analysis with the leave-one-out approach.

Figure S3. Sensitivity analysis including only studies with the Impella pump.

Figure S4. Subanalysis including studies that adopted different definitions of infarct size.

Figure S5. Subanalysis according to the time of reperfusion.

Figure S6. Meta-regression using the year of publication as a covariate.

Figure S7. Funnel plots.

Table S1. PRISMA checklist.

Section/topic	#	Checklist item	Reported on page #
**TITLE**			
Title	1	Identify the report as a systematic review, meta-analysis, or both.	1
**ABSTRACT**			
Structured summary	2	Provide a structured summary including, as applicable: background; objectives; data sources; study eligibility criteria, participants, and interventions; study appraisal and synthesis methods; results; limitations; conclusions and implications of key findings; systematic review registration number.	2
**INTRODUCTION**			
Rationale	3	Describe the rationale for the review in the context of what is already known.	2-3
Objectives	4	Provide an explicit statement of questions being addressed with reference to participants, interventions, comparisons, outcomes, and study design (PICOS).	3
**METHODS**			
Protocol and registration	5	Indicate if a review protocol exists, if and where it can be accessed (e.g., Web address), and, if available, provide registration information including registration number.	CRD42022271799
Eligibility criteria	6	Specify study characteristics (e.g., PICOS, length of follow-up) and report characteristics (e.g., years considered, language, publication status) used as criteria for eligibility, giving rationale.	3
Information sources	7	Describe all information sources (e.g., databases with dates of coverage, contact with study authors to identify additional studies) in the search and date last searched.	3
Search	8	Present full electronic search strategy for at least one database, including any limits used, such that it could be repeated.	Table S2
Study selection	9	State the process for selecting studies (i.e., screening, eligibility, included in systematic review, and, if applicable, included in the meta-analysis).	Table S3
Data collection process	10	Describe method of data extraction from reports (e.g., piloted forms, independently, in duplicate) and any processes for obtaining and confirming data from investigators.	4
Data items	11	List and define all variables for which data were sought (e.g., PICOS, funding sources) and any assumptions and simplifications made.	3-4

Risk of bias in individual studies	12	Describe methods used for assessing risk of bias of individual studies (including specification of whether this was done at the study or outcome level), and how this information is to be used in any data synthesis.	3-4
Summary measures	13	State the principal summary measures (e.g., risk ratio, difference in means).	4
Synthesis of results	14	Describe the methods of handling data and combining results of studies, if done, including measures of consistency (e.g., I^2) for each meta-analysis.	5-6

Table S2. Full electronic search in MEDLINE/Pubmed database through 10 July 2020.

Research	Query	Items Found
1	Left ventricular unloading	1596
2	Myocardial infarction AND left ventricular unloading	273
3	Mechanical circulatory support AND left ventricular unloading	228
4	Mechanical preconditioning	924
5	Primary unloading	617

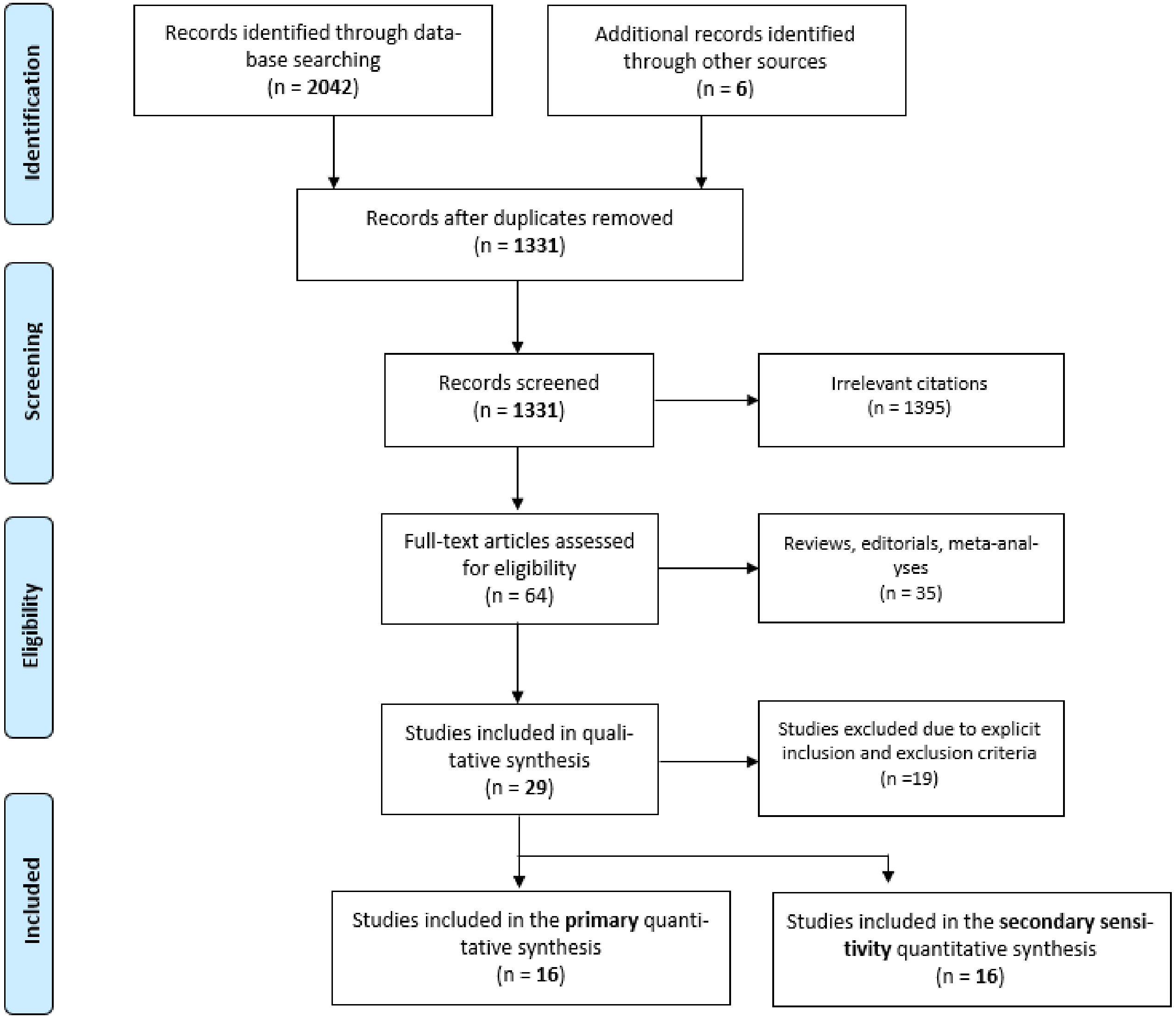

Figure S1. PRISMA flowchart.

Table S3. Bias assessment.

Study	Random allocation sequence	Similar animals	Allocation (algorithm) concealment	Random housing	Blinding of investigators and/or caretakers	Random animal selection for outcome assessment	Blinding of outcome assessor	Incomplete data addressed	Free of selective reporting	Free of other bias	Total scores for each study		
											High	Low	Unclear
**Achour 2005 [17]**	Low	Unclear	Unclear	Unclear	High	Unclear	High	Low	Unclear	Low	**2**	**3**	**5**
**Briceno 2019 [18]**	High	Low	High	Unclear	Low	Unclear	Low	Low	Unclear	Low	**2**	**5**	**3**
**Esposito 2018 [5]**	High	Low	High	Unclear	High	Unclear	High	Low	Unclear	Low	**4**	**3**	**3**
**Kapur 2015 [20]**	Low	Low	Unclear	Unclear	High	Unclear	High	Low	Unclear	Low	**2**	**4**	**4**
**LeDoux 2018 [22]**	High	Unclear	High	Unclear	High	High	High	Low	Unclear	Low	**5**	**2**	**3**
**Meyns 2003 [23]**	High	Low	High	Unclear	High	Unclear	High	Low	Unclear	Low	**4**	**3**	**3**

**Catinella 1983 [19]**	High	Low	High	Unclear	High	Unclear	High	Low	Unclear	Low	**4**	**3**	**3**
**Ko 2020 [21]**	High	Low	High	Unclear	High	Unclear	High	Low	Unclear	Low	**4**	**3**	**3**
**Yoshitake 2012 [26]**	High	Low	High	Unclear	High	Unclear	High	Low	Unclear	Low	**4**	**3**	**3**
**Sunagawa 2018 [24]**	High	Low	High	Low	High	Unclear	High	Low	Unclear	Low	**5**	**3**	**2**
**Tamareille 2008 [25]**	High	Low	High	Unclear	High	Unclear	High	Low	Unclear	Low	**4**	**3**	**3**

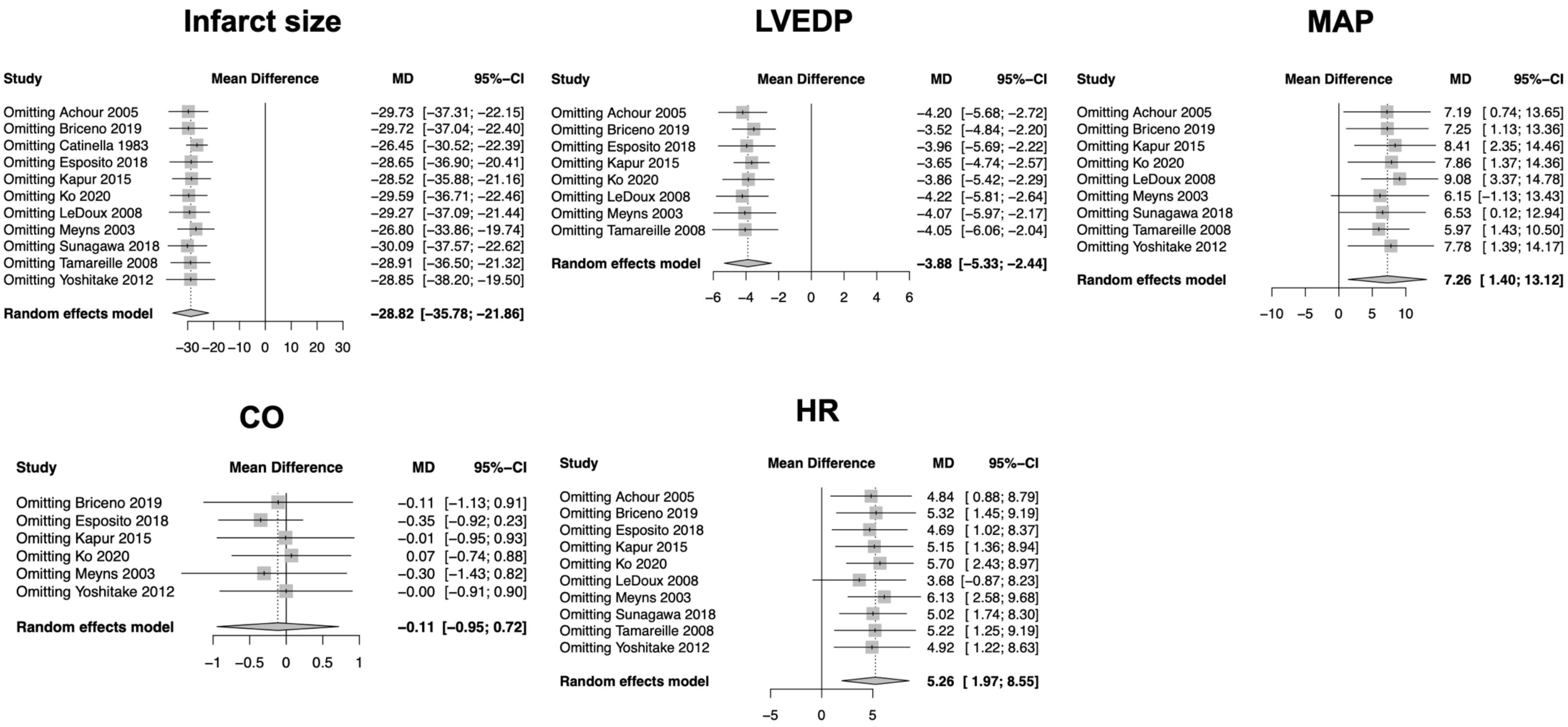

Figure S2. Sensitivity analysis with the leave-one-out approach. References: Achour et al [17], Briceno et al. [18], Esposito et al. [5], Kapur et al. [20], LeDoux et al. [22], Meyns et al. [23], Catinella et al. [19], Ko et al. [21], Yoshitake et al. [26], Sunagawa et al. [24], Tamareille et al. [25].

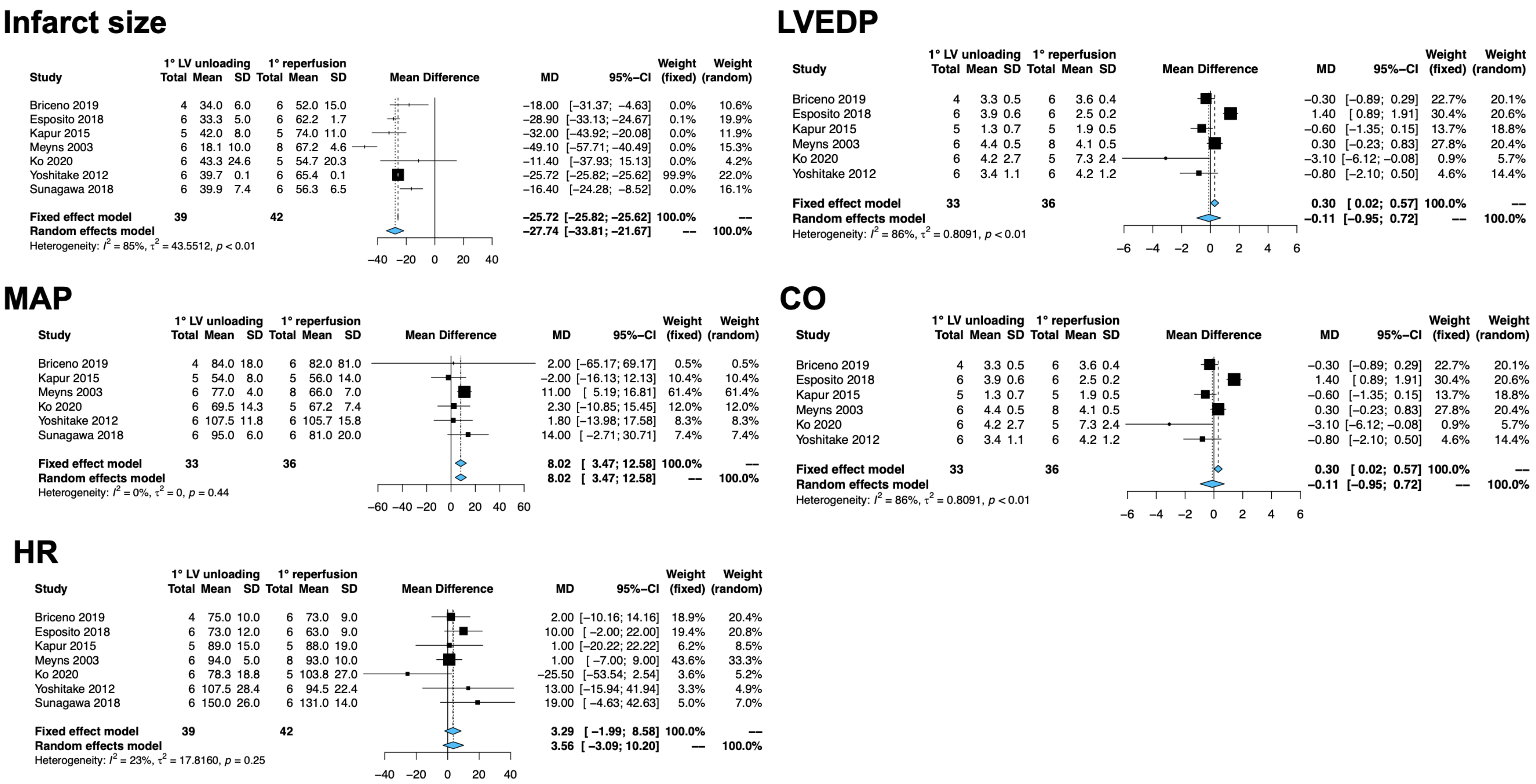

Figure S3. Sensitivity analysis including only studies with the Impella pump. References: Briceno et al. [18], Esposito et al. [5], Kapur et al. [20], Meyns et al. [23], Ko et al. [21], Yoshitake et al. [26], Sunagawa et al. [24].

Study	1° LV unloading Total	Mean	SD	1° reperfusion Total	Mean	SD	MD	95%-CI	Weight (fixed)	Weight (random)
**IS_AAR = 1**										
Achour 2005	8	34.7	4.6	8	54.6	9.2	−19.89	[−27.04; −12.74]	0.0%	6.6%
Briceno 2019	4	34.0	6.0	6	52.0	15.0	−18.00	[−31.37; −4.63]	0.0%	5.3%
Esposito 2018	6	33.3	5.0	6	62.2	1.7	−28.90	[−33.13; −24.67]	0.1%	7.1%
Kapur 2015	5	42.0	8.0	5	74.0	11.0	−32.00	[−43.92; −20.08]	0.0%	5.7%
LeDoux 2008	6	20.9	5.1	6	44.9	4.8	−24.00	[−29.60; −18.40]	0.0%	6.9%
Meyns 2003	6	18.1	10.0	8	67.2	4.6	−49.10	[−57.71; −40.49]	0.0%	6.4%
Catinella 1983	10	21.8	4.6	11	73.7	4.3	−51.90	[−55.72; −48.08]	0.1%	7.1%
Ko 2020	6	43.3	24.6	5	54.7	20.3	−11.40	[−37.93; 15.13]	0.0%	3.0%
Yoshitake 2012	6	39.7	0.1	6	65.4	0.1	−25.72	[−25.82; −25.62]	99.5%	7.3%
Sunagawa 2018	6	39.9	7.4	6	56.3	6.5	−16.40	[−24.28; −8.52]	0.0%	6.5%
Tamareille 2008	6	24.1	6.5	6	51.6	6.6	−27.49	[−34.90; −20.08]	0.0%	6.6%
**Fixed effect model**	**69**			**73**			**−25.74**	**[−25.84; −25.64]**	**99.7%**	**--**
**Random effects model**							**−28.82**	**[−35.78; −21.86]**	**--**	**68.5%**
Heterogeneity: $I^2 = 96\%$, $\tau^2 = 116.6401$, $p < 0.01$										
Test for effect in subgroup (fixed effect): $z = -503.79$ ($p = 0$)										
Test for effect in subgroup (random effects): $z = -8.12$ ($p < 0.01$)										
**IS_AAR = 0**										
Kapur et al. 2013	4	28.0	7.0	4	49.0	14.0	−21.00	[−36.34; −5.66]	0.0%	4.9%
Saku et al. 2018	5	2.1	1.6	5	16.3	2.6	−14.20	[−16.88; −11.52]	0.1%	7.2%
Sun et al. 2015	6	18.1	4.8	6	35.3	6.2	−17.20	[−23.47; −10.93]	0.0%	6.8%
Hashton et al. 1979	3	22.9	2.8	3	38.4	11.2	−15.50	[−28.56; −2.44]	0.0%	5.4%
Wouters et al. 1993	6	11.8	3.2	6	12.8	3.2	−1.00	[−4.62; 2.62]	0.1%	7.1%
**Fixed effect model**	**24**			**24**			**−10.64**	**[−12.64; −8.65]**	**0.3%**	**--**
**Random effects model**							**−12.73**	**[−20.70; −4.77]**	**--**	**31.5%**
Heterogeneity: $I^2 = 90\%$, $\tau^2 = 64.2329$, $p < 0.01$										
Test for effect in subgroup (fixed effect): $z = -10.46$ ($p < 0.01$)										
Test for effect in subgroup (random effects): $z = -3.13$ ($p < 0.01$)										
**Fixed effect model**	**93**			**97**			**−25.70**	**[−25.80; −25.60]**	**100.0%**	**--**
**Random effects model**							**−23.86**	**[−29.79; −17.92]**	**--**	**100.0%**

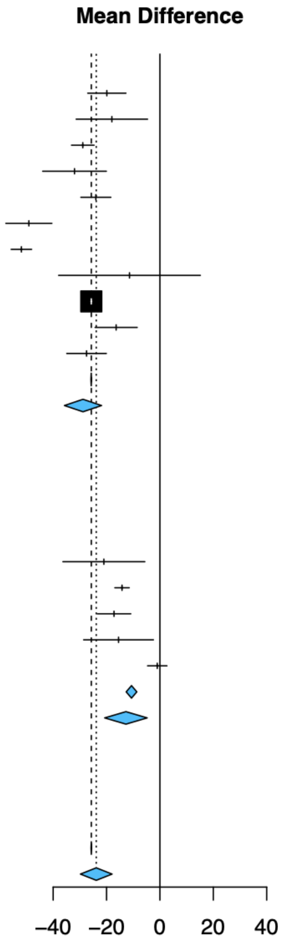

Heterogeneity: $I^2 = 97\%$, $\tau^2 = 124.8207$, $p < 0.01$
Residual heterogeneity: $I^2 = 95\%$, $p < 0.01$
Test for overall effect (fixed effect): $z = -503.68$ ($p = 0$)
Test for overall effect (random effects): $z = -7.88$ ($p < 0.01$)
Test for subgroup differences (fixed effect): $\chi^2_1 = 219.54$, df = 1 ($p < 0.01$)
Test for subgroup differences (random effects): $\chi^2_1 = 8.88$, df = 1 ($p < 0.01$)

Figure S4. Subanalysis including studies that adopted different definitions of infarct size. References: Achour et al [17], Briceno et al. [18], Esposito et al. [5], Hashton et al. [16], Kapur et al. [20], Kapur et al. [6], LeDoux et al. [22], Meyns et al. [23], Catinella et al. [19], Ko et al. [21], Yoshitake et al. [26], Saku et al. [14], Sun et al. [13], Sunagawa et al. [24], Tamareille et al. [25], Wouters et al. [15].

Study	1° LV unloading Total	1° LV unloading Mean	1° LV unloading SD	1° reperfusion Total	1° reperfusion Mean	1° reperfusion SD	MD	95%−CI	Weight (fixed)	Weight (random)
**Delayed_reperfusion = 0**										
Achour 2005	8	34.7	4.6	8	54.6	9.2	−19.89	[−27.04; −12.74]	0.0%	9.7%
Briceno 2019	4	34.0	6.0	6	52.0	15.0	−18.00	[−31.37; −4.63]	0.0%	7.7%
LeDoux 2008	6	20.9	5.1	6	44.9	4.8	−24.00	[−29.60; −18.40]	0.0%	10.1%
Meyns 2003	6	18.1	10.0	8	67.2	4.6	−49.10	[−57.71; −40.49]	0.0%	9.3%
Catinella 1983	10	21.8	4.6	11	73.7	4.3	−51.90	[−55.72; −48.08]	0.1%	10.5%
Yoshitake 2012	6	39.7	0.1	6	65.4	0.1	−25.72	[−25.82; −25.62]	99.8%	10.8%
Sunagawa 2018	6	39.9	7.4	6	56.3	6.5	−16.40	[−24.28; −8.52]	0.0%	9.5%
Tamareille 2008	6	24.1	6.5	6	51.6	6.6	−27.49	[−34.90; −20.08]	0.0%	9.6%
**Fixed effect model**	**52**			**57**			**−25.74**	**[−25.84; −25.64]**	**99.9%**	**--**
**Random effects model**							**−29.34**	**[−38.46; −20.22]**	**--**	**77.2%**
Heterogeneity: $I^2 = 97\%$, $\tau^2 = 158.9556$, $p < 0.01$										
Test for effect in subgroup (fixed effect): $z = -503.59$ ($p = 0$)										
Test for effect in subgroup (random effects): $z = -6.31$ ($p < 0.01$)										
**Delayed_reperfusion = 1**										
Esposito 2018	6	33.3	5.0	6	62.2	1.7	−28.90	[−33.13; −24.67]	0.1%	10.4%
Kapur 2015	5	42.0	8.0	5	74.0	11.0	−32.00	[−43.92; −20.08]	0.0%	8.2%
Ko 2020	6	43.3	24.6	5	54.7	20.3	−11.40	[−37.93; 15.13]	0.0%	4.2%
**Fixed effect model**	**17**			**16**			**−28.85**	**[−32.79; −24.91]**	**0.1%**	**--**
**Random effects model**							**−28.85**	**[−32.79; −24.91]**	**--**	**22.8%**
Heterogeneity: $I^2 = 0\%$, $\tau^2 = 0$, $p = 0.38$										
Test for effect in subgroup (fixed effect): $z = -14.36$ ($p < 0.01$)										
Test for effect in subgroup (random effects): $z = -14.36$ ($p < 0.01$)										
**Fixed effect model**	**69**			**73**			**−25.74**	**[−25.84; −25.64]**	**100.0%**	**--**
**Random effects model**							**−28.82**	**[−35.78; −21.86]**	**--**	**100.0%**

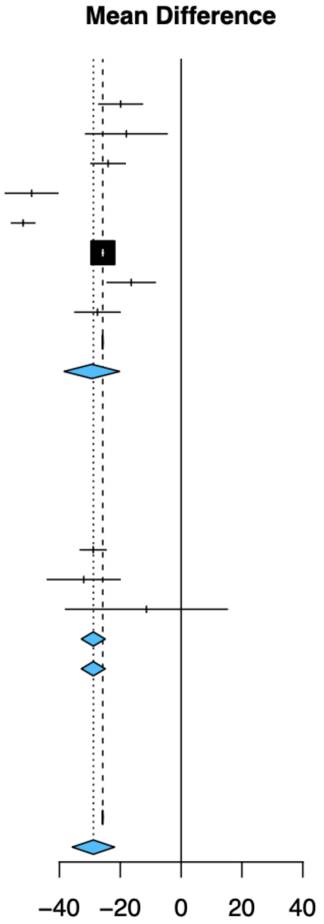

Heterogeneity: $I^2 = 96\%$, $\tau^2 = 116.6401$, $p < 0.01$
Residual heterogeneity: $I^2 = 96\%$, $p < 0.01$
Test for overall effect (fixed effect): $z = -503.79$ ($p = 0$)
Test for overall effect (random effects): $z = -8.12$ ($p < 0.01$)
Test for subgroup differences (fixed effect): $\chi^2_1 = 2.40$, df = 1 ($p = 0.12$)
Test for subgroup differences (random effects): $\chi^2_1 = 0.01$, df = 1 ($p = 0.92$)

Figure S5. Subanalysis according to the time of reperfusion.

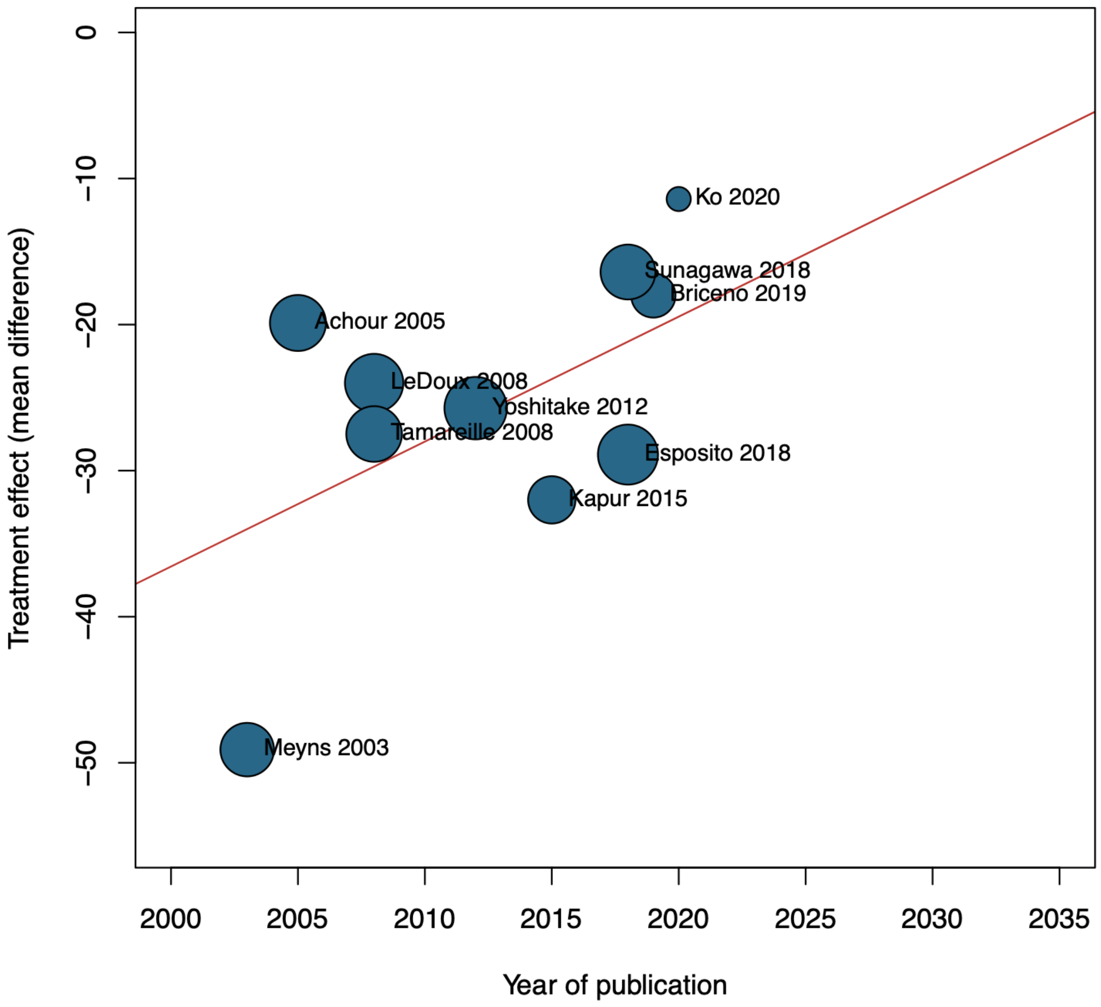

Figure S6. Meta-regression using the year of publication as a covariate. References: Achour et al [17], Briceno et al. [18], Esposito et al. [5], Kapur et al. [20], LeDoux et al. [22], Meyns et al. [23], Ko et al. [21], Yoshitake et al. [26], Sunagawa et al. [24], Tamareille et al. [25].

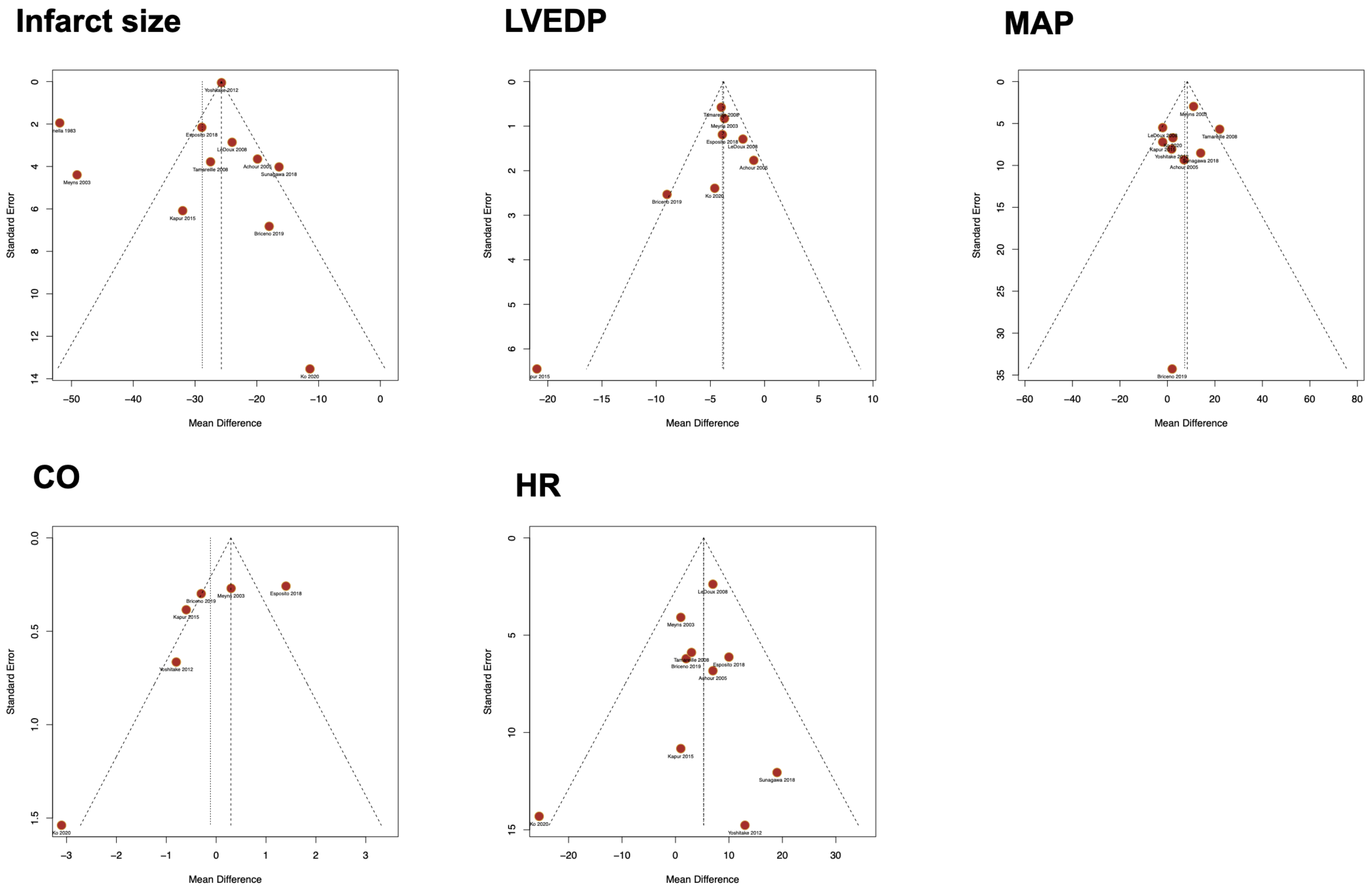

Figure S7. Funnel plots. References: Achour et al [17], Briceno et al. [18], Esposito et al. [5], Kapur et al. [20], LeDoux et al. [22], Meyns et al. [23], Catinella et al. [19], Ko et al. [21], Yoshitake et al. [26], Sunagawa et al. [24], Tamareille et al. [25].
